# Quality in care homes: How wearable devices and social network analysis might help

**DOI:** 10.1371/journal.pone.0302478

**Published:** 2024-05-15

**Authors:** Carl Thompson, Adam Gordon, Kishwer Khaliq, Amrit Daffu-O’Reilly, Thomas Willis, Catherine Noakes, Karen Spilsbury

**Affiliations:** 1 School of Healthcare, University of Leeds, Leeds, West Yorkshire, United Kingdom; 2 Division of Medical Sciences and Graduate Entry Medicine, University of Nottingham, Derby, Derbyshire, United Kingdom; 3 School of Civil Engineering, University of Leeds, Leeds, West Yorkshire, United Kingdom; 4 Leeds Institute of Clinical Trials Research, University of Leeds, Leeds, West Yorkshire, United Kingdom; University of Rwanda College of Medicine and Health Sciences, RWANDA

## Abstract

Social network analysis can support quality improvement in care homes but traditional approaches to social network analysis are not always feasible in care homes. Recalling contacts and movements in a home is difficult for residents and staff and documentary and other sources of individual contacts can be unreliable. Bluetooth enabled wearable devices are a potential means of generating reliable, trustworthy, social network data in care home communities. In this paper, we explore the empirical, theoretical and real-world potential and difficulties in using Bluetooth enabled wearables with residents and staff in care homes for quality improvement. We demonstrate, for the first time, that a relatively simple system built around the Internet of Things, Bluetooth enabled wearables for residents and staff and passive location devices (the CONTACT intervention) can capture social networks and data in homes, enabling social network analysis, measures, statistics and visualisations. Unexpected variations in social network measures and patterns are surfaced, alongside “uncomfortable” information concerning staff time spent with residents. We show how technology might also help identify those most in need of social contact in a home. The possibilities of technology-enabled social network analysis must be balanced against the implementation-related challenges associated with introducing innovations in complex social systems such as care homes. Behavioural challenges notwithstanding, we argue that armed with social network information, care home staff could better tailor, plan and evaluate the effects of quality improvement with the sub-communities that make up a care home community.

## Introduction

Imagine two residents living in a UK care home (with nursing) in the same dementia community: Registered Nurses (RNs) always on duty, home rated “good” by the regulator, 20% turnover of staff and an in-house programme of continuing professional development for staff. Both residents have similar needs: help with mobilising, nutrition and hydration, continence care and impaired communication. Each day one resident usually gets seen by the same carer 5 times in an eight-hour shift for around 6 minutes in their bedroom and 30 mins sat next to the same resident in a communal dining room for meals. The second resident usually has contact with three carers for 15 minutes on 10 occasions, in their bedroom, with an hour in the home’s lounge and 45 minutes mealtime sat with three other residents.

Who is likely receiving the higher quality care? Do these patterns of contact provide information that helps judge the quality of care and life in homes?

Care homes (long-term care facilities, nursing homes, residential homes) are a societal response to increasing numbers of older people in many countries. Almost half a million older people reside in some form of care home in the UK alone [1]. Care home quality varies and homes sometimes face public scrutiny and criticism. The quality of care for older people reflects societal values and priorities. Not providing high-quality care to older members of society violates their dignity and autonomy [2]. High-quality care that meets one’s needs and expectations is a fundamental human right.

Defining and describing quality is conceptually, empirically and methodologically challenging. Conceptually, quality relates to both quality of care (what staff do, how they do it, the environment in which care is delivered) and quality of life (how care impacts on residents’ function, physical and psychological needs, autonomy, and dignity) [3]. Spilsbury argues this distinction is artificial: quality of care is a determinant of quality of life in care homes [4].

Empirically, operationalising quality (with its inherently subjective components) into indicators and associated measures, will always result in “missed” dimensions and aspects of experience. Indicator-based quality assessments are also open to misinterpretation if indicators are not viewed in the contexts in which they are applied [4]. Whilst some approaches to quality and assessment—for example, Donabedian’s decomposition of the concept into structural, process and outcome based dimensions—encourage a more comprehensive and logical basis for measuring and assuring quality, the problem of subjectivity (what matters to residents) remains.

Methodologically, there is no single reliable method with high internal and external validity for evaluating care home quality. Why? because care homes are complex social systems, made up of diverse interacting and interdependent groups. People exercise their agency within structures and processes that influence the values, beliefs and behaviours of other people in the (social) system, and how it is experienced by those who live and work in it [5]. A home’s quality is both a property (something it has) and a function (something it creates). Philosophically, there is no quality inherent in objects, quality is subjective and includes the emotional experience an object provides [6]. For Pirsig, for something to have quality needs those judging and creating it to be intimately involved in it:

*“Working well*, *caring*, *is to become a part of the process*, *to achieve an inner peace of mind*.*”*

[6].

Pirsig’s conceptual approach to quality is particularly applicable to care homes; because (high quality) care—dressing, washing, communicating–involves co-production [7].

Spilsbury et al. [8] developed a logic model of the staffing-quality relationship in homes which explains, empirically and theoretically, what works, why and how, [8] as well as the interactions between the constituent structures, processes, and outcomes [9]. Understanding how to meet the needs and preferences of residents in care homes efficiently (given rising costs and demand) given the available care workforce to promote quality is a societal priority. Haunch and colleagues used a realist synthesis of varying approaches to quality to uncover the “generative mechanism(s)” behind quality in the context in which it is co-produced [10]. They outline a series of context-mechanism-outcome configurations to increase quality enhancing behaviours in staff. The mechanism underpinning each context-mechanism-outcome configuration is “relationships”.

Relationships are a key component in quality promoting behaviours generally [11] and specifically. For example, implementing specific nutritional guidelines more effectively by generally harnessing relationships and the power of interactions in home environments [12]. They are a key element in mid-level theories of (implicit) quality such as relationship-centred care [13]. Thus, quality in care homes is relational: generated within and by the interactions between residents and staff and the size and strength of their social networks. Interactions with other people are a crucial part of a quality care environment [14, 15]. Without adequate social connections, residents risk social isolation, loneliness, and diminished health states. Something seen so vividly during the COVID-19 pandemic [16]. Inadequate social connections make providing quality care more difficult. Improving the quality of care and outcomes for residents, means addressing social networks in care homes, designing and implementing strategies to promote social connections and support among residents and staff and harnessing the power in social networks and network-focused interventions.

If social networks could be described reliably and efficiently, the metrics and statistics of social network analysis (SNA) could be used to inform the design, implementation and monitoring of structured change programs, policies and practices aimed at accelerating change/improvement using homes’ own social networks [17, 18].

Valente presents a taxonomy of four network intervention approaches: (i) recruiting individuals based on their characteristics (in the network), (ii) using subgroups in a network, (iii) changing networks by adding/removing ties or nodes, and (iv) encouraging more interactions. Whilst network interventions theoretically promise changing behaviour in health and social care environments such interventions are rarely tested empirically. Saatchi and colleagues [18] found only 4 examples of SNA used as the basis for an intervention (in healthcare) and just 74 examples of SNA simply describing networks; figures that hadn’t changed much in ten years [17]. In their review of social-professional networks in long term care settings for people with dementia, Van Beek et al. found only 9 examples of SNA used to describe networks in long term care, with just a single study suggesting networks improve care (for example, staff taking more time with residents or observed friendliness in staff-resident interactions) in measurable ways [19].

One reason why social networks are not leveraged more often, is because describing social networks in care homes reliably and efficiently is challenging. Traditionally, social network analytic methods rely on recalling interactions using interview, documentary and/or roster/survey data, or they may draw on data derived from social media or other network data [20].

Researchers have employed SNA in and with care homes. Spilsbury and colleagues [8], and Sales [21] and Cott [22] all used roster-based approaches and restricted analyses to staff. Roster or recall based approaches are inappropriate for care homes. Many (>75%) of residents may have memory problems, and staff may struggle to recall historic contacts in an environment where contact is both frequent and unavoidable. Solutions exploiting the Bluetooth capabilities of smartphones have been proposed and widely implemented in non-care home contexts (c.f. national contact tracing efforts as part of the COVID-19 pandemic) [23]. But smartphone solutions are unsuited to care homes: few residents use them, staff may be discouraged from using them and the risk of false positives is high [24]. Wearables that exploit Bluetooth and other communications technology (low frequency wide area networks/LoRaWAN and the Internet of Things/IOT) offer a promising alternative for capturing social network data. Bluetooth enabled (BLE) wearable based approaches have shown promise for examining proximity networks in healthcare [25] and informing models of infection in long term care [24].

**Fig 1:**
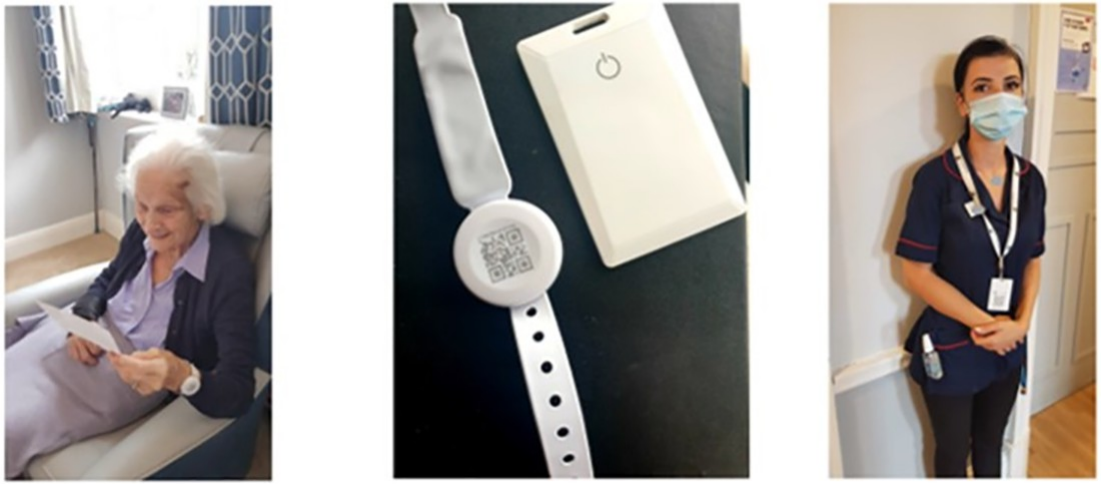
CONTACT digital wearables, reprinted from Thompson, C.A., Daffu-O’Reilly, A., Willis, T. et al. ‘Smart’ BLE wearables for digital contact tracing in care homes during the COVID-19 pandemic—a process evaluation of the CONTACT feasibility study. Implement Sci Commun 4, 155 (2023), under a CC BY license, with permission from Springer Nature, original copyright 2023.

The empirical data and analysis for this paper comes from the CONTACT study [26]. CONTACT was a feasibility investigation to determine the viability of BLE wearables in fob (worn as a watch or brooch) or card (attached to a lanyard) forms (see Fig 1), stationary location markers and data transmission from care homes to a clinical trials unit for analysis. CONTACT’s intervention had three components: (i) contact data from staff, resident, and visitor BLE wearables and location markers; (ii) structured feedback as monthly reports and in response to newly detected COVID-19 cases; and (iii) support for the care homes to interpret the analysis in reports for use in their IPC planning, decisions and judgements.

We have published CONTACT’s full feasibility, process evaluation, and technical evaluations elsewhere [27, 28]. In this paper we explore the value of BLE wearable-enabled SNA for quality improvement in care homes. Significantly, as it’s for the first time in a care home context, we highlight those ways technology, data and social network analysis can be used to realise the potential in knowledge of social networks in care homes. The paper is offered as a “proof of principle” with illustrative examples and the strengths and limitations described in the context of care homes.

## Methods

The CONTACT feasibility study [29] was part of a planned cluster randomised trial of the CONTACT tracing and feedback intervention; ISRCTN registration: 11204126. The study sites were four care homes in North and West Yorkshire, England, UK. recruitment was between September 20^th^ 2021 to 28^th^ November 2021 and data were collected between 28^th^ November 2021 and February 28^th^ 2022.

Tables 1 and 2 outline the homes and each home’s sub communities.

**Table 1:** The four care homes as context.

Home^$^	Type	CQC rating	Ownership	Maximum capacity	Number of staff	Number of residents	Number of residents with dementia	Device type issued
Home 1: Quillton	Residential and nursing	Requires Improvement	For profit, some private equity backing	45	77	35	17	Fob
Home 2: Newchurch	Nursing	Good	For profit (owner manager)	15	21	15	2	Card
Home 3: Fordlandia	Nursing	Good	For profit (owner manager)	28	37	23	5	Fob
Home 4: Brownhall	Residential and nursing	Good	For profit, some private equity	102	120 (70*)	87 (37*)	25	Fob

* The devices were worn in two units of the three in the home;

^**$**^Homes are pseudonyms

**Table 2:** Home community roles.

		Role			
		Agency (n)	Resident (n)	Staff (n)	Total (n)
**Care Home**	Quillton	-	35	77	99
	Newchurch	-	12	19	31
	Fordlandia	3	15	32	50
	Brownhall*	2	37	69	108

**Fig 2:**
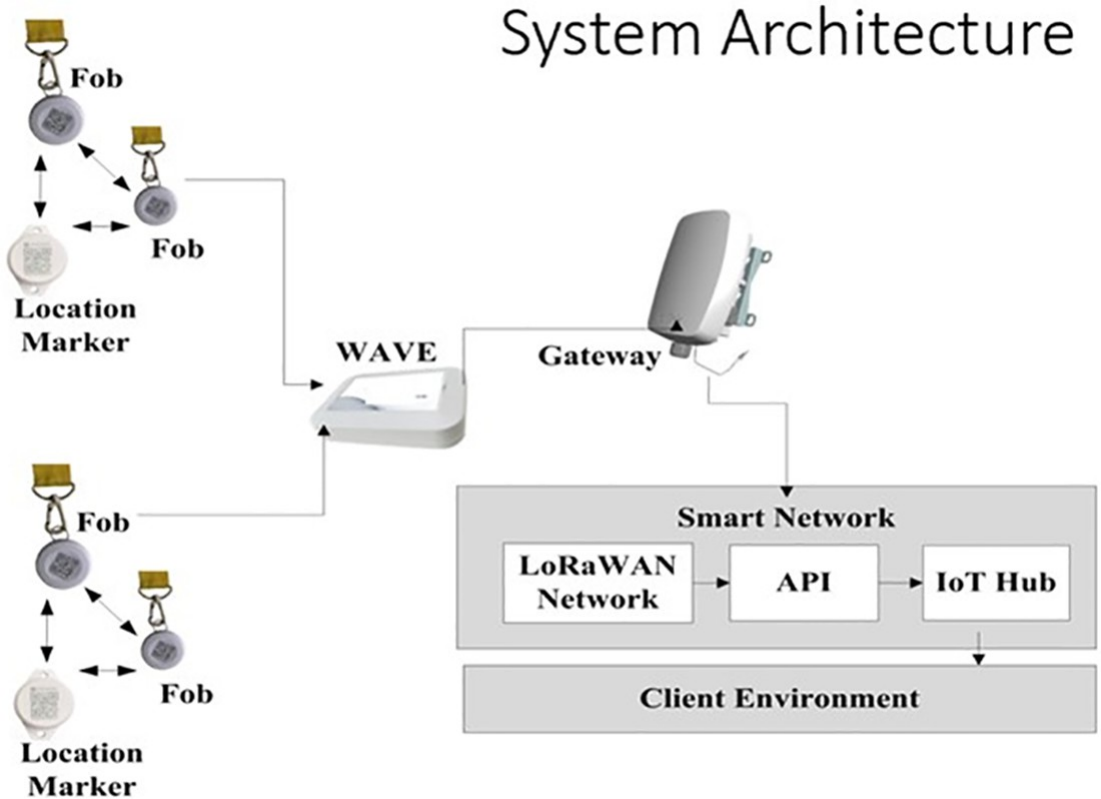
System architecture needed to generate contact based social network data, reprinted from Kishwer Abdul Khaliq, Catherine Noakes, Andrew H. Kemp, Carl Thompson & the CONTACT trial team (2023) evaluating the performance of wearable devices for contact tracing in care home environments, journal of occupational and environmental hygiene, 20:10, 468–479, under a CC BY license, with permission from Taylor Francis, original copyright 2023.

MICROSHARE.inc provided system hardware [30]. Each homes setup was similar, only the numbers of wearable devices and location markers (a function of home’s size) differed. See Fig 2 for system architecture.

Personal Bluetooth Low Energy (BLE) wearable devices continuously scan for and record other wearables or location markers. A wave [31] scanner collects contact information and transmits data from wearables and location markers to a telecommunications gateway using a Long Range Wide Area Network (LoRaWAN). Anonymized data from Microshare’s cloud server was exported to our university secure data infrastructure for analysis.

Consent was individual, in writing and we made adjustments (using consultees and nominees) to accommodate residents without capacity to make their own decisions (full details available at https://njl-admin.nihr.ac.uk/document/download/2035361). Consenting participants wore a BLE wearable in the home. Wearables and static location markers had a unique identifier which homes could use to de-anonymise device wearers for contact and location tracing. Our study protocol is published elsewhere [26] and ethical approval for the study was sought and granted by CONTACT study by the UK Health Research Authority REC: 294390.

Each home’s social network data comprised:

- device-device IDs (who had contact with whom)
- date and time stamp of the contact (when did the contact happen) which meant we could calculate:
  - frequency of contacts in a given time-slot
  - the length of individual and mean number of contacts
- where the contact occurred (the nearest location marker)
- the signal strength (Received Signal Strength Indicator or RSSI) between wearable devices; a proxy for how close the devices were to each other.

### Analysis

For each home we calculated counts, means and standard deviations of interactions (contacts) between devices (people); median and interquartile range of edge weights (relationship strength) and the median duration (in minutes) and interquartile range of each interaction (contact); counts, means and 5^th^ and 95^th^ percentiles of degree centrality for each node (person) and weighted degree centrality (relationship strength-adjusted connectivity). We calculated means and standard deviation of signal strength between devices (signal strength/RSSI of <-75 indicates physical distance of less than 1.5 metres). The SNA analyses and metrics presented in Table 3 were based on established and standard procedures [32]. We used the modularity procedures of Blondel et al. [33] to delineate sub networks. All social network analysis was conducted in UCINET [34] (version 6); visualisation of networks produced using GEPHI version 0.10. [35] with other quantitative analysis and graphs produced via IBM SPSS V26 [36].

**Table 3:** SNA metrics and potential contribution to quality improvement in care homes.

Measure	Definition	Potential application to quality improvement interventions in care homes.
Degree Centrality	The number of connections a node (individual) has in the network	Identify influential staff or stakeholders in the care home; Target training or information dissemination to maximize impact on quality improvement
Closeness Centrality	The mean distance between a node and all other nodes in the network	Identify key individuals who can quickly disseminate information or best practices; Address isolation or siloed communication within the care home
Betweenness Centrality	The number of shortest paths that pass through a node	Identify potential bottlenecks in information flow or decision-making; Address gaps in communication or power dynamics within the care home
Eigenvector Centrality	The extent to which a node is connected to other well-connected nodes	Identify influential staff or stakeholders that have connections to other influential individuals; Leverage relationships for collaborative quality improvement efforts
Density	The proportion of potential connections in the network that are present: the ratio between the edges present in a network and the maximum number of edges that the network can contain	Assess overall communication and collaboration within the care home; Identify areas where additional connections or relationships can be fostered for quality improvement
Clustering Coefficient	The extent to which a node’s neighbours are also connected to each other	Assess the cohesiveness of care home teams or departments; Strengthen collaboration and knowledge sharing among staff for quality improvement
Network Centralization	The degree to which the network is organized around a central node or group of nodes	Identify central nodes that can be targeted for change management initiatives; Recognize potential power imbalances or centralization that may hinder quality improvement
Network Modularity	The degree to which the network is divided into subgroups or communities	Identify existing subgroups within the care home; Leverage subgroups for tailored quality improvement interventions or initiatives
Reciprocity	The extent to which connections between nodes are mutual (in a directed network)	Assess the balance and fairness of communication and collaboration among staff; Foster more equitable and inclusive relationships for quality improvement
Assortativity	The tendency of nodes to connect with other nodes that have similar attributes (e.g., age, role, etc.)	Identify potential biases or cliques within the care home; Promote diversity and inclusion within teams for broader perspectives on quality improvement

## Results and discussion

### Identifying communities within a home

**Fig 3:**
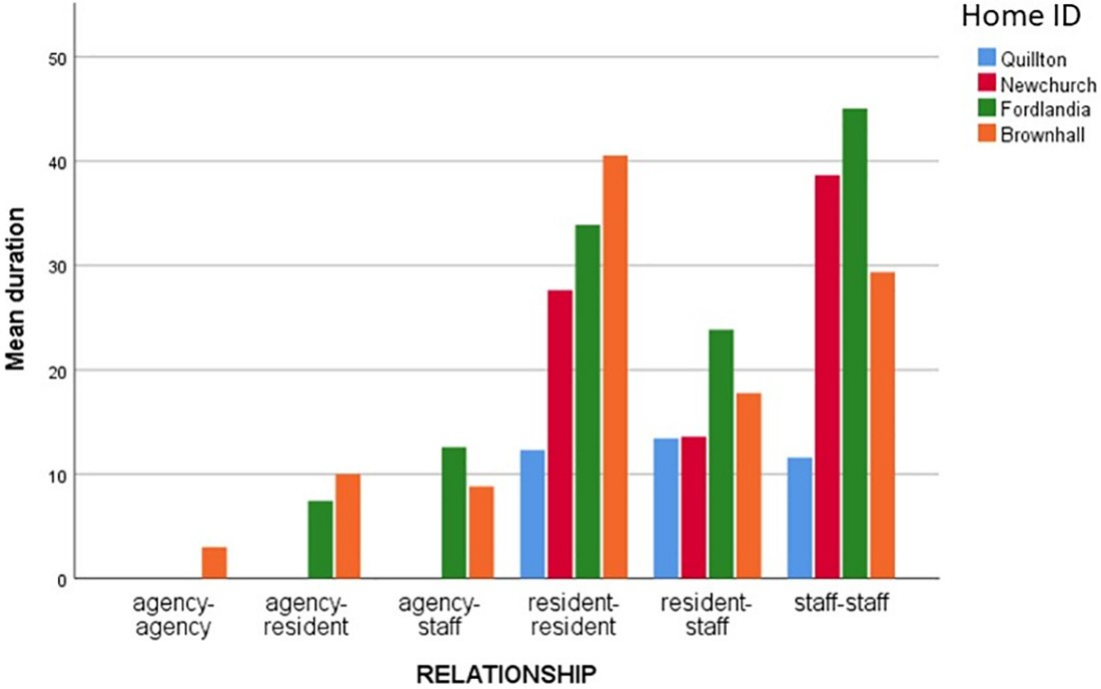
Interaction time (duration) by home and homegroup.

**Fig 4:**
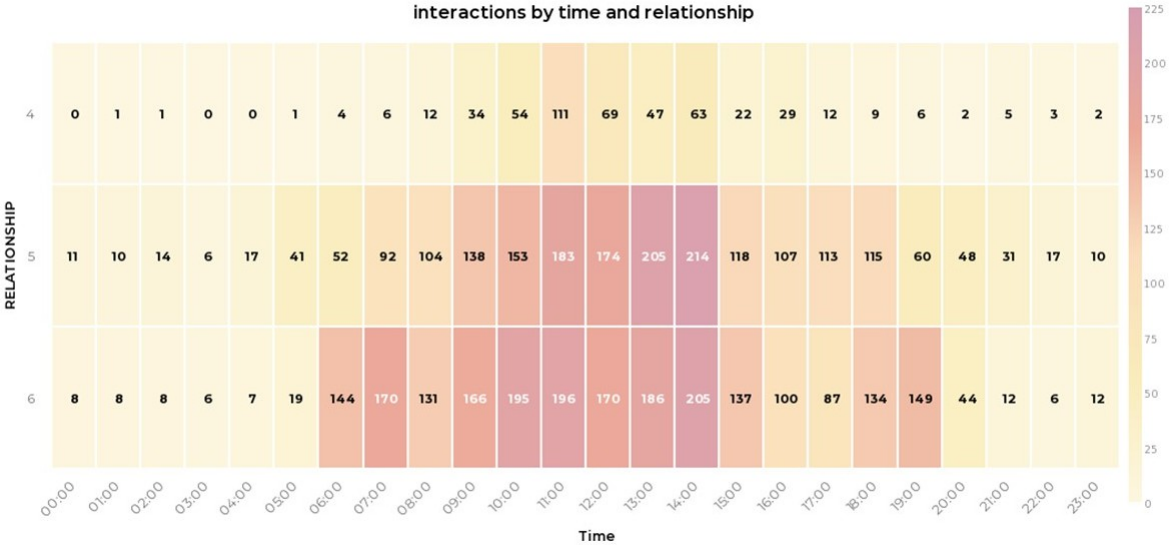
Heat map of interaction frequency. (legend: 4 = res-res; 5 = res-staff; 6 = staff-staff).

**Fig 5:**
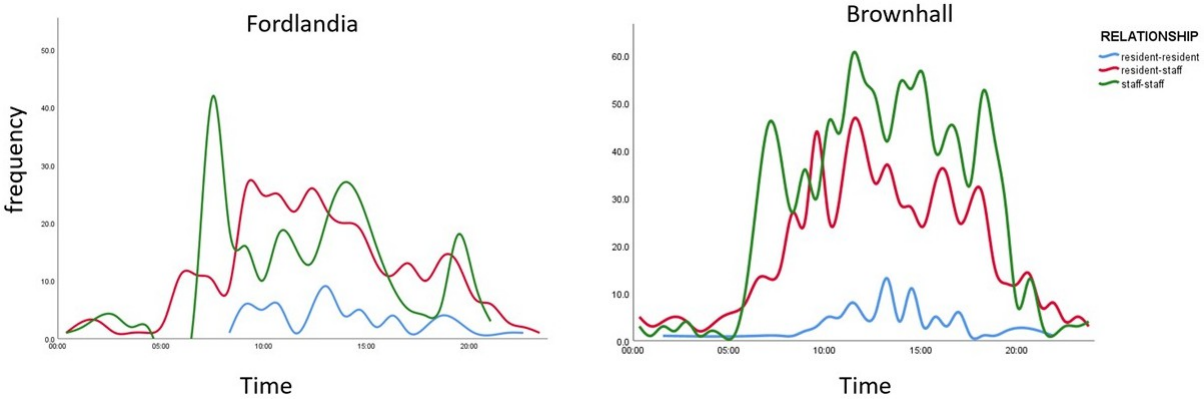
Interactions over time by relationship.

**Fig 6:**
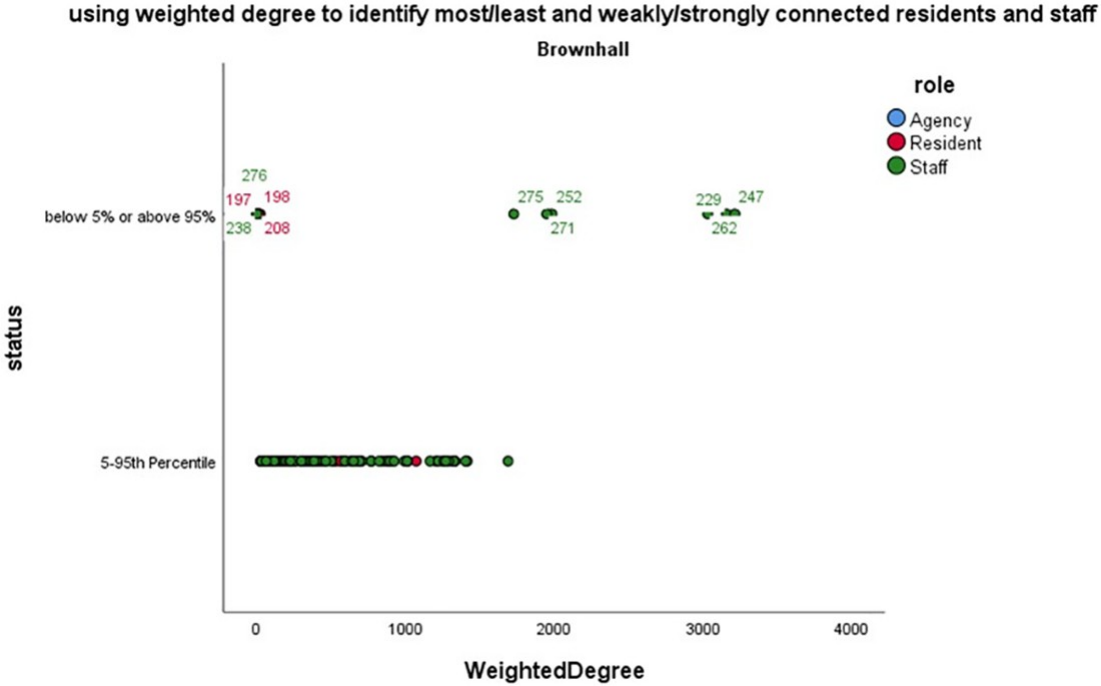
Identifying the most (and least) connected community members using number and strength of interactions.

**Fig 7:**
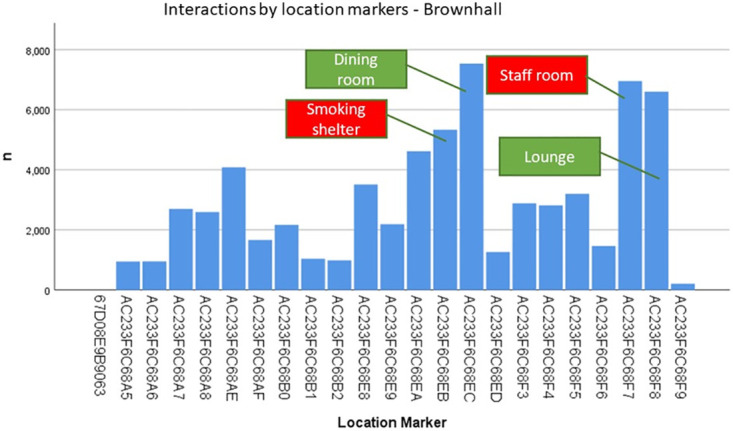
Frequency of interactions by home locations in Brownhall.

Analysing the data generated by the BLE wearables highlights dimensions of quality that those planning, implementing or evaluating care may wish to consider. Illustrative examples include, reducing variability in duration (see Table 4 and Fig 3) and concentration of interactions in the home day (see Figs 4 and 5); extending the size of social networks for individuals (see Table 5 and Fig 6); reducing the between and within home differences in staff-resident and staff-staff interactions (Fig 3); ensuring that a location’s potential to enable interaction is maximised (see Fig 7).

**Table 4:** Strength of interactions and duration by home.

Home	Weight (median)	Interquartile Range	Duration (Median minutes)	Interquartile Range
Quillton	18	88	9	4
Newchurch	79	155	9	13
Fordlandia	13	36	13	25
Brownhall	5	12	8	8

**Table 5:** Mean connections and strength adjusted connections by care home and role.

		n	Mean Degree centrality	5^th^ Percentile	95% Percentile	Mean Weighted degrees	5^th^ Percentile	95% Percentile
Quillton	Agency	0	.	.	.	.	.	.
	Resident	35	39	13	60	2665	90	6067
	Staff	64	39	9	70	1803	18	5838
Newchurch	Agency	0	.	.	.	.	.	.
	Resident	12	26	21	28	4507	1596	7292
	Staff	19	26	1	30	2634	1	4930
Fordlandia	Agency	3	5	1	12	14	1	40
	Resident	15	30	17	38	729	156	1544
	Staff	32	29	4	40	549	4	1243
Brownhall	Agency	2	27	9	45	365	157	572
	Resident	37	24	6	49	306	20	885
	Staff	69	40	13	70	794	64	1984

**Fig 8:**
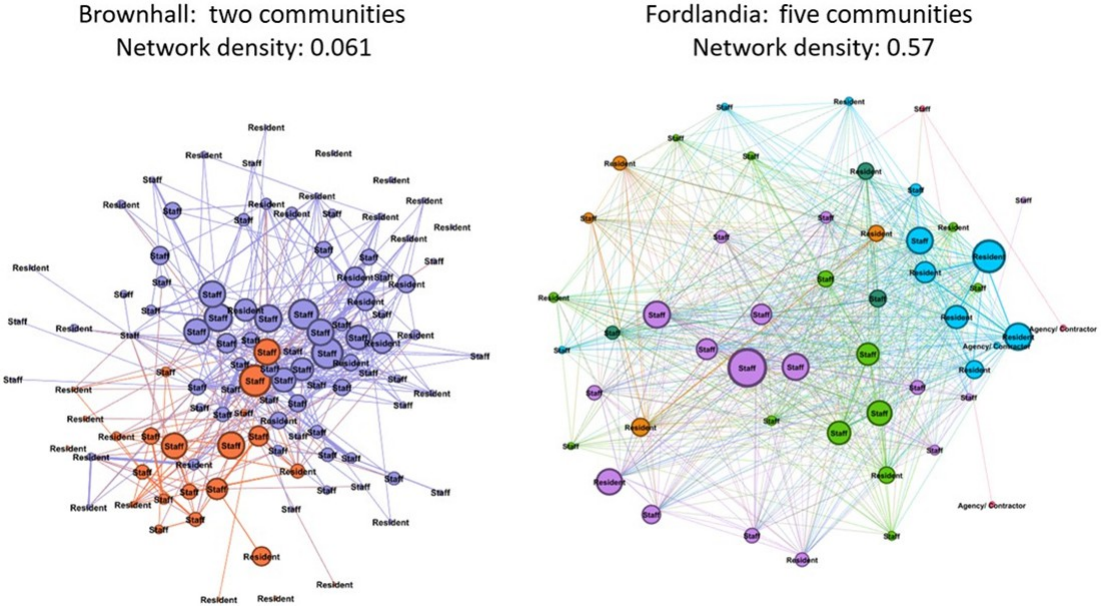
Larger homes don’t always mean more subcommunities (node size indicates degree centrality [bigger = more] and edge width the frequency of interactions [edge weight; thicker = stronger]).

Fig 8 shows that homes can be conceptualised and viewed as subcommunities, based on the numbers and strength of relationships between community members. Brownhall is a bigger home and has two distinct, but less dense, (density = 0.06) subcommunities. Fordlandia is smaller but more cohesive (density = 0.57), with five more tightly bound subcommunities.

### What does interaction look like?

There were 204,087 interactions between people in the four homes over two months.

Just 2% (n = 4893) of interactions were over two minutes, but more than sixty five percent (67.4%) of these involved staff (Table 6); double the proportion of resident interactions (32%). Staff explanations included “doubling up” (caring in pairs). CONTACT’s BLE wearable data would have counted situations in which one resident had contact with two staff as two interactions: one for each member of staff. The “doubling up” argument does not hold, staff had more interaction with other staff than with residents.

**Table 6:** Interactions of >2minutes by roles.

			Role			Total
			Agency	Resident	Staff	
Role	Agency	n	1	14	24	39
	Resident		5	493	972	1470
	Staff		23	1061	2300	3384
		Total	29	1568	3296	4893
		% of Total	0.6%	32.0%	67.4%	100.0%

The quantity of interactions provides only partial clues to their role as generative mechanisms for quality [10]. As important are behaviours occurring whilst interacting. Behaviours occurring *within* interactions between staff and residents are not always positive [15, 37, 38]. Indeed, Siette and colleagues found lower quality of life in residents with more staff interactions [15]. This may be explained by the greater needs (and thus lower quality of life) of residents with more staff contact. Observational data however reveals “contact” does not always equate to meaningful engagement, activity, or communication. Edwards and colleagues found the most commonly observed behaviour in contact with residents was *no response* to residents (63% of their n = 1708 observations with 20 residents). Supportively engaging residents only occurred in 12% of their observations [37].

Interactions between residents is linked to better quality of life [39]. In our data only one of the four homes had roughly equivalent amounts of interaction between the variations of staff and resident groups (see Fig 3, Quillton).

Fig 6 highlights staff and residents with the strongest (and weakest) connections to their communities–strength being contact frequency and intensity. This may be appropriate (for example, in an infection control context), but for an environment in which “homeliness” is the goal, it may not. These structures in home communities are a key driver and context for quality for theorists such as Avis Donabedian [9]. Previous descriptions of long term care social structures are based on rosters [40] or interviews [39]. BLE wearable-based approaches may generate a more reliable picture of social networks.

Whilst communal areas such as dining rooms and lounges were the main focal point for interactions, Fig 7 reveals interactions in staff and smoking rooms. A manager wishing to foster a more resident-centred approach to quality may wish to decrease the use of these settings, and increase the time that staff spend in communal areas with residents.

### Patterns of interaction (opportunities for quality promoting behaviours) in a home day?

Staff deploying quality-promoting behaviours require sufficient interactions with the right number of residents. But they also require the time to enact those behaviours in sensitive, reflective and supportive ways [10]. Median interaction duration in CONTACT’s four homes varied from 8 to 13 minutes (see Table 4). Mallidou and colleagues found around a third (35%) of interactions were between 1 and 3 minutes, with half focused on “direct care” (feeding, helping to the toilet etc.) [41]. To our knowledge, researchers have not studied patterns of interaction in the 24-hour activity cycle of care homes. Our results suggest quality-generating opportunities exist at different times of the day. Fig 8 shows resident-resident interactions have a modest increase from mid-morning to early afternoon, but staff-resident and staff-staff interactions increase in frequency and duration markedly between 10am and 3pm. Anyone seeking opportunities to promote quality enhancing behaviours may find that targeting the behaviours that happen in this window reaches the most people whilst maximising chances for reflective learning. Conversely, a manager might question how quieter parts of the day are used to promote quality in homes. Fig 5 shows residents have relatively few interactions with other residents, but also that staff-staff interaction is greatest at the beginning, middle and end of a typical 8am-8pm day shift–perhaps because of structured routines such as handovers between shifts. It also shows staff-staff interactions in these two homes are sometimes independent of contact with residents; something managers may wish to understand and address.

### The small world of care

Interaction is a necessary, but not sufficient, condition for quality [10]. Assuming managers and others interested in quality are satisfied with the quantity and duration of contacts in their home, Table 7 shows residents and staff have relatively small numbers of *unique* interactions: 2% and 3% of their interactions respectively. The size and variety of resident’s social networks influences quality of life [39, 42]. Casey et al. [39] showed how forming new friendships in care homes is difficult, how residents depend on staff to facilitate relationships, and how residents’ behavioural, physical and psychological traits can build or reduce social capital in homes. Social capital—for example, participating in groups–can improve resident function [43]. As others have found [37], staff may underexploit opportunities for fostering interactions to increase residents’ social networks and leverage relational aspects of care quality; for example, during mealtimes and nutrition [12].

**Table 7:** Unique interactions and all interactions by home and role.

		Individuals (n)	Mean Unique Interactions—n, (% of all interactions)	SD	Mean Interactions—n	SD
role	Agency	5	14 (9%)	18	154	242
	Resident	99	31 (2%)	15	1713	2111
	Staff	184	36 (3%)	17	1292	1615

### Limitations

Social network analysis from BLE wearables provides only partial information for promoting quality in care homes. SNA metrics require interpretation *in context*, something that can only come from staff knowing their community. Providing more SNA information may not help. For example, a manager asking for a more granular algorithm to identify (more) than Brownhall’s (two) subnetworks, may be confronted with greater detail but fewer economies of scale in improvement approaches. Tailoring quality interventions to sub-networks’ may also increase costs [44].

Syntheses of research on quality in care homes highlights the importance of reciprocity in community relationships [10]. Reciprocity is linked to openness in information sharing and receptiveness, and can help build confidence and trust in the workforce. We have previously looked at information and advice flows in directed care home networks [8]. We were able to describe reciprocity in information and advice giving and receiving in the care home networks and link this to care home quality [8].

CONTACT’s home network data were undirected. Using knowledge from BLE wearables to explore reciprocity requires knowledge of participants’ influence on each other and who influences them. We didn’t have this knowledge. Casey and colleagues’ [39] highlight an important trade-off of resident-focused SNA: BLE wearable data may generate internally valid picture of structures, but is unable to handle directionality in relationships and thus, reciprocity. Without collecting more data, important (for quality) relational variables such as emotional support or strength of ties can only be *inferred*, from interactions, timings, frequency etc [39].

Two other noteworthy limitations of BLE wearables relate to implementation and ethics. When we first asked staff to wear BLE devices, it was in a pre-vaccination, very uncertain, COVID-19 pandemic context. When BLE wearables were eventually deployed, it was in a post-vaccination, more certain, context. Staff were aware their movements could be revealed (to managers), and that patterns of contact (i.e. work) and time in specific locations (such as smoking shelters, residents’ or staff rooms) would be made visible. Some staff felt threatened. Some homes (Fordlandia) had almost total compliance, others (Brownhall) were (at best) ambivalent and (at worst) rapidly disengaged. Using BLE wearables for sustained quality improvement, like any complex intervention, requires a well-conceived, co-developed, plan informed by implementation science [45].

BLE wearables like all trackable devices raise ethical challenges around privacy and consent–particularly for those residents without mental health capacity. Our procedures were particularly robust, with the formal assessment, and use of personal and nominated consultees needed for a research study. See our parent study protocol for full details [26]. Similar safeguards would be required however in a non-research context if ethical deployment of wearables for network analysis is to be assured.

## Conclusion

BLE devices worn by care home residents and staff can generate useful data for quality improvement. Movement, interaction times, durations, and composition can help in the planning, implementation and evaluation of initiatives to combat well known problems in the care sector: isolated residents; unequal workloads; limited interaction between staff and residents and routinised—rather than reflexive and responsive–care. Sub-communities can be identified and quality improvement tailored to norms and network characteristics.

SNA could be used alongside established QI approaches such as statistical process control (SPC), or to explore how the dynamic nature of date and time-stamped network data might help examine the impact of time on home life in sophisticated and transparent ways, less prone to self-report or performance biases. Environmental aspects of care home quality (such as air quality, thermal comfort and humidity) and the influence of social networks might also be incorporated into SNA. There are undoubted challenges associated with BLE-enabled SNA for QI in care homes, but the potential is significant. We are hopeful technical advances and changing post-pandemic attitudes in and to care homes will help care providers make the most of this potentially valuable technology as they strive to enhance quality for people working and living in care homes.

## References

[1] Care homes and estimating the self-funding population, England—Office for National Statistics. [cited 1 Jun 2023]. https://www.ons.gov.uk/peoplepopulationandcommunity/healthandsocialcare/socialcare/articles/carehomesandestimatingtheselffundingpopulationengland/2021to2022

[2] Ahmed PK, Machold S. The Quality and Ethics Connection: Toward Virtuous Organizations. Total Qual Manag Bus Excell. 2004;15: 527–545. doi: 10.1080/1478336042000183604

[3] Malley J, Fernández J-L. Measuring Quality in Social Care Services: Theory and Practice. Ann Public Coop Econ. 2010;81: 559–582. doi: 10.1111/j.1467-8292.2010.00422.x

[4] Spilsbury K, Hewitt C, Stirk L, Bowman C. The relationship between nurse staffing and quality of care in nursing homes: A systematic review. Int J Nurs Stud. 2011;48: 732–750. doi: 10.1016/j.ijnurstu.2011.02.014 21397229

[5] Anderson RA, Issel LM, McDaniel RRJ. Nursing Homes as Complex Adaptive Systems: Relationship Between Management Practice and Resident Outcomes. Nurs Res. 2003;52: 12. doi: 10.1097/00006199-200301000-00003 12552171 PMC1993902

[6] Pirsig RM. Zen and the art of motorcycle maintenance. Toronto; New York: Bantam, [1975] [©1974]; 1975. https://search.library.wisc.edu/catalog/999867832502121

[7] Towers A-M, Smith N, Allan S, Vadean F, Collins G, Rand S, et al. Care home residents’ quality of life and its association with CQC ratings and workforce issues: the MiCareHQ mixed-methods study. Southampton (UK): NIHR Journals Library; 2021. http://www.ncbi.nlm.nih.gov/books/NBK574833/34723450

[8] Spilsbury K, Charlwood A, Thompson C, Haunch K, Valizade D, Devi R, et al. Understanding the Staffing Relationship to Quality in care homes: the StaRQ mixed-methods study. NIHR Heath Services Delivery and Research; 2022. 10.3310/GWTT8143 38634535

[9] Donabedian A. The Definition of Quality and Approaches to Its Assessment. Health Administration Press; 1980.

[10] Haunch K, Thompson C, Arthur A, Edwards P, Goodman C, Hanratty B, et al. Understanding the staff behaviours that promote quality for older people living in long term care facilities: A realist review. Int J Nurs Stud. 2021/03/15 ed. 2021;117: 103905. doi: 10.1016/j.ijnurstu.2021.103905 33714766

[11] Nolan M, Davies S, Brown J. Transitions in care homes: towards relationship‐centred care using the ‘Senses Framework.’ Qual Ageing Older Adults. 2006;7: 5–14. doi: 10.1108/14717794200600015

[12] Keller HH, Syed S, Dakkak H, Wu SA, Volkert D. Reimagining Nutrition Care and Mealtimes in Long-Term Care. J Am Med Dir Assoc. 2022;23: 253–260.e1. doi: 10.1016/j.jamda.2021.12.021 34986411

[13] Dewar B, Nolan M. Caring about caring: Developing a model to implement compassionate relationship centred care in an older people care setting. Int J Nurs Stud. 2013;50: 1247–1258. doi: 10.1016/j.ijnurstu.2013.01.008 23427893

[14] Sion KYJ, Verbeek H, Zwakhalen SMG, Odekerken-Schröder G, Schols JMGA, Hamers JPH. Themes Related to Experienced Quality of Care in Nursing Homes From the Resident’s Perspective: A Systematic Literature Review and Thematic Synthesis. Gerontol Geriatr Med. 2020;6: 2333721420931964. doi: 10.1177/2333721420931964 32637461 PMC7318818

[15] Siette J, Dodds L, Surian D, Prgomet M, Dunn A, Westbrook J. Social interactions and quality of life of residents in aged care facilities: A multi-methods study. PLOS ONE. 2022;17: e0273412. doi: 10.1371/journal.pone.0273412 36037181 PMC9423621

[16] Suárez-González A, Rajagopalan J, Livingston G, Alladi S. The effect of COVID-19 isolation measures on the cognition and mental health of people living with dementia: A rapid systematic review of one year of quantitative evidence. eClinicalMedicine. 2021;39. doi: 10.1016/j.eclinm.2021.101047 34386758 PMC8342894

[17] Chambers D, Wilson P, Thompson C, Harden M. Social network analysis in healthcare settings: a systematic scoping review. PloS One. 2012;7: e41911. doi: 10.1371/journal.pone.0041911 22870261 PMC3411695

[18] Saatchi AG, Pallotti F, Sullivan P. Network approaches and interventions in healthcare settings: A systematic scoping review. PLOS ONE. 2023;18: e0282050. doi: 10.1371/journal.pone.0282050 36821554 PMC9949682

[19] van Beek APA, Wagner C, Frijters DHM, Ribbe MW, Groenewegen PP. The ties that bind? Social networks of nursing staff and staff’s behaviour towards residents with dementia. Soc Netw. 2013;35: 347–356. doi: 10.1016/j.socnet.2013.03.006

[20] Scott J. Social network analysis: A handbook. Thousand Oaks, CA, US: Sage Publications, Inc; 1991. pp. x, 210.

[21] Sales AE, Estabrooks CA, Valente TW. The impact of social networks on knowledge transfer in long-term care facilities: Protocol for a study. Implement Sci. 2010;5: 49. doi: 10.1186/1748-5908-5-49 20573254 PMC2900220

[22] Cott C. “We decide, you carry it out”: A social network analysis of multidisciplinary long-term care teams. Soc Sci Med. 1997;45: 1411–1421. doi: 10.1016/s0277-9536(97)00066-x 9351158

[23] Anglemyer A, Moore TH, Parker L, Chambers T, Grady A, Chiu K, et al. Digital contact tracing technologies in epidemics: a rapid review. Cochrane Database Syst Rev. 2021/01/28 ed. 2020;8: CD013699. doi: 10.1002/14651858.CD013699 33502000 PMC8241885

[24] Wilmink G, Summer I, Marsyla D, Sukhu S, Grote J, Zobel G, et al. Real-Time Digital Contact Tracing: Development of a System to Control COVID-19 Outbreaks in Nursing Homes and Long-Term Care Facilities. JMIR Public Health Surveill. 2020;6: e20828. doi: 10.2196/20828 32745013 PMC7451111

[25] Curtis SJ, Rathnayaka A, Wu F, Al Mamun A, Spiers C, Bingham G, et al. Feasibility of Bluetooth Low Energy wearable tags to quantify healthcare worker proximity networks and patient close contact: A pilot study. Infect Dis Health. 2022;27: 66–70. doi: 10.1016/j.idh.2021.10.004 34810151 PMC8963530

[26] CONtact TrAcing in Care homes using digital Technology (CONTACT)—A pragmatic cluster randomised controlled trial, cost-effectiveness evaluation and theory-informed process evaluation.—NIHR Funding and Awards. [cited 25 Apr 2023]. https://fundingawards.nihr.ac.uk/award/NIHR132197

[27] Khaliq KA, Noakes C, Kemp AH, Thompson C. Evaluating the performance of wearable devices for contact tracing in care home environments. J Occup Environ Hyg. 2023;in press. doi: 10.1080/15459624.2023.2241522 37540215

[28] Thompson CA, Daffu-O’Reilly A, Willis T, Gordon A, Noakes C, Khaliq K, et al. ‘Smart’ BLE wearables for digital contact tracing in care homes during the COVID-19 pandemic—a process evaluation of the CONTACT feasibility study. Implement Sci Commun. 2023;4: 155. doi: 10.1186/s43058-023-00533-0 38049924 PMC10694939

[29] Eldridge SM, Lancaster GA, Campbell MJ, Thabane L, Hopewell S, Coleman CL, et al. Defining Feasibility and Pilot Studies in Preparation for Randomised Controlled Trials: Development of a Conceptual Framework. PLOS ONE. 2016;11: e0150205. doi: 10.1371/journal.pone.0150205 26978655 PMC4792418

[30] Lustig M. Universal Contact Tracing Solution. In: Microshare.io—Unleash the Data [Internet]. [cited 7 Jun 2023]. https://www.microshare.io/universal-contract-tracing-solution/

[31] Wanesy Wave. In: Kerlink [Internet]. [cited 3 Aug 2023]. https://www.kerlink.com/wanesy-wave/

[32] Borgatti SP, Everett MG, Johnson JC. Analyzing Social Networks. SAGE; 2018.

[33] Blondel VD, Guillaume J-L, Lambiotte R, Lefebvre E. Fast unfolding of communities in large networks. J Stat Mech Theory Exp. 2008;2008: P10008. doi: 10.1088/1742-5468/2008/10/P10008

[34] Borgatti SP, Everett MG, Freeman LC. Ucinet for Windows: Software for social network analysis. Harv MA Anal Technol. 2002;6: 12–15.

[35] Gephi—The Open Graph Viz Platform. [cited 27 Apr 2023]. https://gephi.org/

[36] SPSS Statistics—Overview. 19 Apr 2023 [cited 27 Apr 2023]. https://www.ibm.com/products/spss-statistics

[37] Edwards H, Gaskill D, Sanders F, Forster E, Morrison P, Fleming R, et al. Resident-staff interactions: a challenge for quality residential aged care. Australas J Ageing. 2003;22: 31–37. doi: 10.1111/j.1741-6612.2003.tb00460.x

[38] Saldert C, Bartonek-Åhman H, Bloch S. Interaction between Nursing Staff and Residents with Aphasia in Long-Term Care: A Mixed Method Case Study. Nurs Res Pract. 2018;2018: e9418692. doi: 10.1155/2018/9418692 30631596 PMC6304643

[39] Casey A-NS, Low L-F, Jeon Y-H, Brodaty H. Residents Perceptions of Friendship and Positive Social Networks Within a Nursing Home. The Gerontologist. 2016;56: 855–867. doi: 10.1093/geront/gnv146 26603182

[40] Ayalon L, Yahav I, Lesser O. From a Bird’s Eye View: Whole Social Networks in Adult Day Care Centers and Continuing Care Retirement Communities. Innov Aging. 2018;2: igy024. doi: 10.1093/geroni/igy024 30480144 PMC6176959

[41] Mallidou AA, Cummings GG, Schalm C, Estabrooks CA. Health care aides use of time in a residential long-term care unit: A time and motion study. Int J Nurs Stud. 2013;50: 1229–1239. doi: 10.1016/j.ijnurstu.2012.12.009 23312466

[42] Abbott KM, Pachucki MC. Associations between social network characteristics, cognitive function, and quality of life among residents in a dementia special care unit: A pilot study. Dementia. 2017;16: 1004–1019. doi: 10.1177/1471301216630907 26862130

[43] Andrew MK. Social capital, health, and care home residence among older adults: a secondary analysis of the Health Survey for England 2000. Eur J Ageing. 2005;2: 137–148. doi: 10.1007/s10433-005-0031-8 28794726 PMC5547683

[44] Thompson C, Pulleyblank R, Parrott S, Essex H. The cost-effectiveness of quality improvement projects: a conceptual framework, checklist and online tool for considering the costs and consequences of implementation-based quality improvement. J Eval Clin Pract. 2016;22: 26–30. doi: 10.1111/jep.12421 26201387

[45] Devi R, Martin GP, Banerjee J, Gladman JR, Dening T, Barat A, et al. Sustaining interventions in care homes initiated by quality improvement projects: a qualitative study. BMJ Qual Saf. 2022 [cited 26 May 2023]. doi: 10.1136/bmjqs-2021-014345 35318273 PMC10646854

